# RobOKoD: microbial strain design for (over)production of target compounds

**DOI:** 10.3389/fcell.2015.00017

**Published:** 2015-03-24

**Authors:** Natalie J. Stanford, Pierre Millard, Neil Swainston

**Affiliations:** ^1^Manchester Institute of Biotechnology, University of ManchesterManchester, UK; ^2^School of Computer Science, University of ManchesterManchester, UK; ^3^INSA, UPS, INP, LISBP, Université de ToulouseToulouse, France; ^4^INRA, UMR792, Ingénierie des Systèmes Biologiques et des ProcédésToulouse, France; ^5^Centre National de la Recherche Scientifique, UMR5504Toulouse, France

**Keywords:** synthetic biology, systems biology, metabolic engineering, strain design, constraint-based modeling

## Abstract

Sustainable production of target compounds such as biofuels and high-value chemicals for pharmaceutical, agrochemical, and chemical industries is becoming an increasing priority given their current dependency upon diminishing petrochemical resources. Designing these strains is difficult, with current methods focusing primarily on knocking-out genes, dismissing other vital steps of strain design including the overexpression and dampening of genes. The design predictions from current methods also do not translate well-into successful strains in the laboratory. Here, we introduce RobOKoD (Robust, Overexpression, Knockout and Dampening), a method for predicting strain designs for overproduction of targets. The method uses flux variability analysis to profile each reaction within the system under differing production percentages of target-compound and biomass. Using these profiles, reactions are identified as potential knockout, overexpression, or dampening targets. The identified reactions are ranked according to their suitability, providing flexibility in strain design for users. The software was tested by designing a butanol-producing *Escherichia coli* strain, and was compared against the popular OptKnock and RobustKnock methods. RobOKoD shows favorable design predictions, when predictions from these methods are compared to a successful butanol-producing experimentally-validated strain. Overall RobOKoD provides users with rankings of predicted beneficial genetic interventions with which to support optimized strain design.

## Introduction

The sustainable production of target compounds such as biofuels and high-value chemicals for pharmaceutical, agrochemical, and chemical industries is becoming an increasing priority given their current dependency upon diminishing petrochemical resources. The challenge of producing such compounds from microbial cells straddles both systems and synthetic biology. The development of microbial cell factories first requires a comprehensive understanding of host cell metabolic functions through metabolic model construction, and subsequent *in silico* experimentation, using systems biology methods. This *in silico* experimentation can suggest host cell manipulations that can be applied *in vitro* using synthetic biology techniques, leading to increased production of the target compound (Koide et al., 2009).

Target producing microbial strains are typically designed using combinations of gene manipulations. These manipulations include gene additions (often recombinant genes from other organisms) and removal of genes via knockouts. Furthermore, over-expression or inhibition of host genes can either increase or dampen metabolic flux through the reactions that their expressed proteins catalyze. Successful application of such strategies can be used to overproduce host-native targets (Ng et al., 2012; Li et al., 2014) or produce non-host-native targets (Atsumi et al., 2009; Angermayr et al., 2014; Yuan et al., 2014). Identifying successful gene manipulation combinations has traditionally relied on static network inspection, and experimental trial and error to test the strategies (Varman et al., 2011). This approach is not optimal as it limits the amount of network information that can be used, discounts metabolic complexity, and therefore prevents predictions of less intuitive metabolic modifications (Kitano, 2002).

Through modeling approaches, strain predictions can be improved by taking into account full metabolic complexity during the design phase. Designed strains can also be screened *in silico* before they are engineered and tested in the laboratory. The process involves iterative application of the following steps: (i) characterization of the host metabolic network; (ii) identification of gene additions to bridge native metabolism to the target; (iii) optimization of the modified metabolic network through gene addition, deletion, overexpression or dampening; (iv) trialing successful predictions in the laboratory. This process affords the potential to develop successful strains more cost effectively, and time efficiently. This work focuses on step (iii), which involves elements of network characterization in order to identify suitable optimization strategies.

To characterize the metabolic network, genome-scale models (GEMs) can be used in conjunction with constraint-based techniques. GEMs are computer-analyzable, structured knowledge bases of genes, proteins, and metabolites present within a given organism (Thiele and Palsson, 2010). GEMs therefore encode the complexity of host cell metabolism and are available for an increasingly large number of organisms (Büchel et al., 2013). Constraint based techniques, including flux balance analysis (FBA) and flux variability analysis (FVA), provide quantitative predictions of cellular behavior such as metabolic flux patterns and cellular growth rates. These are computed by applying constraints, which can be assigned from experimentally measured nutrient uptake rates (Orth et al., 2010) and intracellular fluxes (Sauer, 2006), or inferred through interpretation of gene expression data (Lee et al., 2012). These predictions provide insights into the metabolic pathways active under different growth conditions (Liao et al., 2011), gene essentiality (Joyce and Palsson, 2008; Dobson et al., 2010; Heavner et al., 2012), and as a result, the fitness optimality of a given strain (Harcombe et al., 2013). More detailed introductions to these techniques can be found in Boxes 1, 2.

**Box 1**

**Figure B1a:**
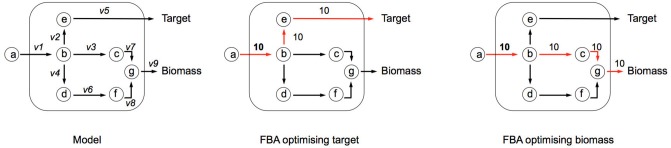
**Illustrating FBA for independent optimisation of target and biomass**.

Flux Balance Analysis (FBA) allows the computation of fluxes, and cellular growth, by using a set of constraints. FBA uses the stoichiometric matrix (*S*), which is a matrix consisting of rows of metabolites (*m*), and columns of reactions (*n*). An example based on the toy network in Figure B1a can be seen in Table B1a. The matrix is usually sparse and filled with positive (negative) coefficients for metabolites produced (consumed) by a reaction. Linear programming is used to compute feasible fluxes (*v*) through the network ensuring that a steady state is satisfied (Equation i), subject to a set of constraints (Equation ii) and optimizing (*Z*) a specific function (Equation iii, where *c* is a vector of weights, typically a vector of zeros with biomass production set to 1). The minimum solutions of Equation (i) are elementary modes, which are minimal sets of enzymes that can operate at steady state, also known as minimal functional units (de Figueiredo et al., 2009). If Equation (i) cannot be satisfied, then FBA cannot be computed on the system.

(i) Sv=0

(ii) $$lb_{i}\leq v_{i}\geq ub_{i},i=1,\ldots ,n\;$$

(iii) $$Z=c^{T}v$$

In the example network below (Figure B1a), *c* is given as an uptake rate of 10 units of metabolite **a**. In the center network *Z* = Target, and in the right-hand network *Z* = Biomass. Reaction bounds are all assigned as *lb*_i_ = 0, *ub_i_* = 1000. Meaning that each reaction through the network is irreversible. Computing FBA for *Z* = Target we get 10 units of flux flowing through *v2* and *v3*, producing *v*__*Target*_ = 10 units. For *Z* = Biomass we get 10 units of flux flowing through *v3*, *v7*, and *v9*, producing *v*__*Biomass*_ = 10 units.

**Table B1a:** **Stoichiometric matrix (S)**.

	***v1***	***v2***	***v3***	***v4***	***v5***	***v6***	***v7***	***v8***	***v9***
a	−1	0	0	0	0	0	0	0	0
b	+1	−1	−1	−1	0	0	0	0	0
c	0	0	+1	0	0	0	−1	0	0
d	0	0	0	+1	0	−1	0	0	0
e	0	+1	0	0	−1	0	0	0	0
f	0	0	0	0	0	+1	0	−1	0
g	0	0	0	0	0	0	+1	+1	−1
bio.	0	0	0	0	0	0	0	0	+1
tar.	0	0	0	0	+1	0	0	0	0

**Box 2**

**Figure B2a:**
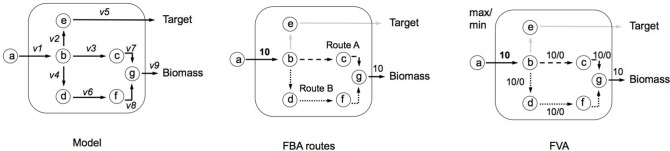
**Illustrating implementation of FVA and how it can be used to identify alternative flux optima**.

Flux Variability Analysis (FVA). Box 1 showed an example of FBA, where a single set of fluxes was identified, which can maximize biomass production (*Z*). It can be seen in the central network of Figure B2a, that this set of fluxes was just one of two possible solutions that could be selected to maximize *Z*—route A and route B. FVA allows us to garner this additional information by identifying the minimum and maximum flux that each reaction can carry (Equation i). FVA can be implemented at the optimal state whereby *y* = 1 (Equation ii), subject to flux constraints for each reaction (Equation iii) as demonstrated in the right-hand network in Figure B2a (Gudmundsson and Thiele, 2010). Here the main information identified is which reactions are interchangeable. It is also common to compute FVA under suboptimal conditions (i.e., *y* = 0.95 as used in RobOKoD), which introduces a small amount of flexibility in the system and reduces the chances of optimal pathways being unrealistic when compared *in vivo*.

(i) $$v_{\text{max}}/v_{\text{min}}v_{i}$$

(ii) $$\gamma Z_{0}\leq c^{\text{T}}v$$

(iii) $$v_{lb}\leq v\leq v_{ub}$$

Optimization of microbial strains is complex, requiring a balance between target production and cell viability (Lo et al., 2013). This makes the problem a multi-objective optimization problem, whereby metabolic flux of cellular growth and target production must be considered simultaneously. Successful optimization strategies therefore include gene modifications (knockouts, overexpression, dampening) which re-route flux toward the target product whilst minimizing the effect on flux toward synthesis of metabolites required for cellular maintenance.

Amongst the more prominent methods used for identifying knockout targets are OptKnock (Burgard et al., 2003) and RobustKnock (Tepper and Shlomi, 2010). OptKnock aims to optimize the maximum flux toward the target product whilst retaining cell viability, using up to five reactions knockouts to generate the strain solution. The method does not take into consideration flux variability, and therefore whilst there may be a reasonable maximal flux yield toward to target product, it is possible that the minimal flux toward the target product could be zero. RobustKnock was developed to improve on this shortcoming, by optimizing the minimal flux toward the target product, again by applying up to five reaction knockouts. Limitations of these methods include the prediction of only a single gene knockout strategy, and also no consideration of over-expression or dampening targets, which are key aspects of successful strain design (Dellomonaco et al., 2011). A complementary method, optGene (later updated to optFlux (Rocha et al., 2010)), can be used for overexpression analysis. Flux Variability Analysis has been used in a number of studies for identifying overexpression targets (Choi et al., 2010; Park et al., 2012), as well as more comprehensive strategies (Pharkya and Maranas, 2006; Feist et al., 2010), although these have not been extensively used. Elementary modes have also been used to identify suitable knockout targets (Ballerstein et al., 2012; von Kamp and Klamt, 2014).

**Figure 1:**
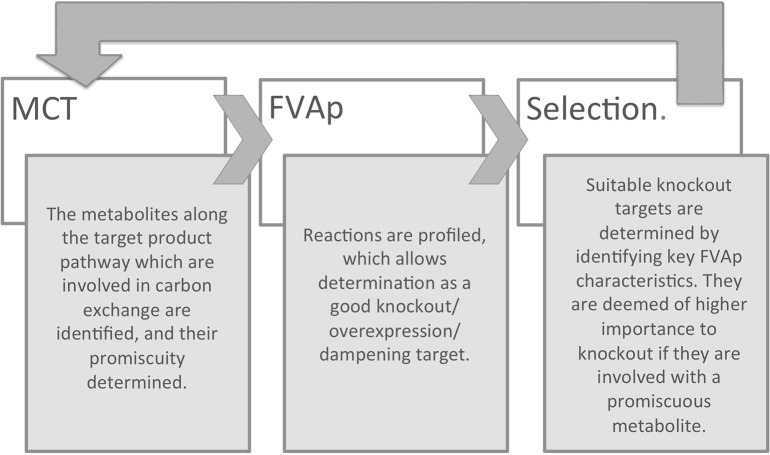
**Workflow of RobOKoD, illustrating the iterative application of the methods MCT and FVAp**.

To integrate the requirements of predicting both knockouts and over-/under-expressions, we introduce RobOKoD (Robust Overexpression, Knockout and Dampening). RobOKoD takes into consideration metabolite centrality and flux variability in order to comprehensively identify potential knockouts and gene over-/under-expressions, ranked by significance, and follow the schematic presented in Figure 1. This ranking is a strength, as it allows for further, manual analysis of the system to be used for strain design.

The performance of RobOKoD was tested against that of OptKnock and RobustKnock in their ability to predict an engineering strategy for production of butanol from *Escherichia coli* using the reverse β-oxidation cycle. The predictions were validated against a successful, experimentally-validated butanol producing strain developed by Dellomonaco et al. (2011).

## Materials and methods

### *Escherichia coli* model

**Figure 2:**
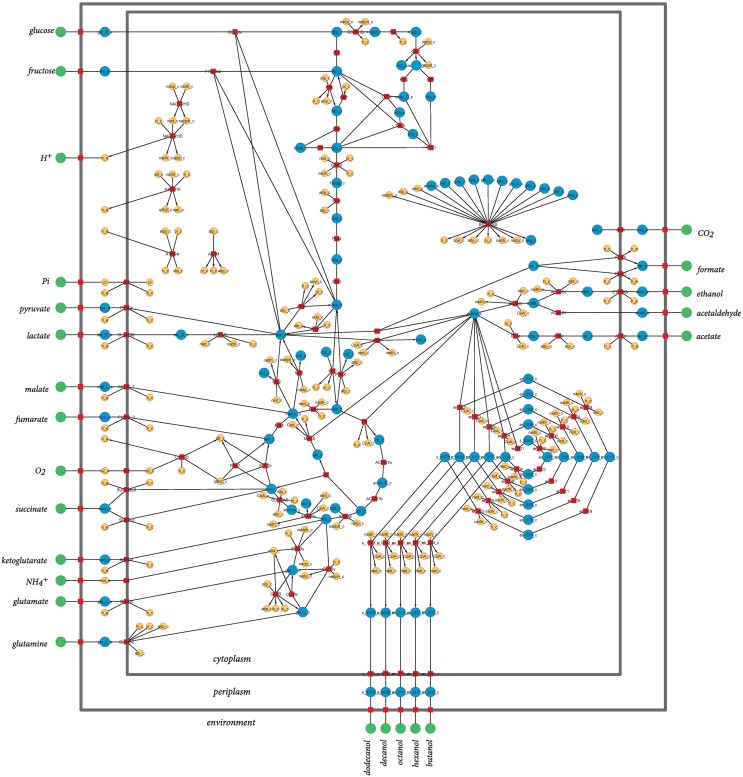
**Graphical representation of the metabolic network of *Escherichia coli* included in iNS142**. Red squares represent reactions, and green, blue, and orange circles represent extracellular metabolites, intracellular metabolites involved in carbon transfers, and intracellular metabolites not involved in carbon transfers, respectively. Directed arcs show irreversible reactions, whereas undirected arcs show reversible reactions. Water is not shown for clarity of the layout.

The model used in this study is a derivation of a core metabolism model derived from the iAF1260 reconstruction of *E. coli* metabolism proposed by Feist et al. (2007). The core metabolism model of 95 native reactions was modified to include the β-oxidation pathway—a total of eight genes catalyzing 30 additional reactions—to produce the model iNS142 (see Table 1). This model contains 142 genes, 125 reactions, and 93 metabolites (Figure 2). The model is available in Supplementary Folder 1 in SBML format (Hucka et al., 2003).

**Table 1:** **Reactions and genes added to the core iAF1260 model to implement the β-oxidation cycle**.

**Reaction**	**Gene(s)**	**EC**
Thiolase	*fadA, fadI*	2.3.1.16
Hydroxyacyl-CoA dehydrogenase	*fadB, fadJ*	1.1.1.35
Enoyl-CoA hydratase	*fadB, fadJ*	4.2.1.17
Enoyl-CoA reductase	*fadE*	1.3.8.1
Alcohol/acetaldehyde dehydrogenase	*frmA, adhP, adhE*	1.1.1.1

### RobOKoD

The RobOKoD method is based on the two following assumptions:

1. To achieve target production, carbon transfer within the network has to be oriented toward pathways that favor the target. Therefore, changes within the network should aim to reduce carbon loss to peripheral pathways.
2. Flux variability of each reaction will differ depending on whether the reaction is important for growth, generating the desired product, both, or neither. Therefore, the functionality of each reaction can be inferred by analyzing its variability.

A simplified schematic of the method based on these two assumptions can be seen in Figure 1 and additional details are given in the next sections. First, a metabolite consumption test (MCT) is applied which computes whether a given metabolite in the target production pathway demonstrates flux loss to biomass production. If flux loss is identified, all reactions that consume that metabolite are flagged as potentially favored targets. Second, flux variability analysis profiling (FVAp) is performed to determine the flux variability of each reaction, at increments of maximum biomass flux and then at increments of maximum target product flux. The profiles of each reaction are used to calculate a score from which the importance of each reaction for growth and target production can be estimated. Finally, MCT and FVAp results are combined to rank potential modifications. Modifications can consist of (i) gene deletions; (ii) changes of environmental conditions; (iii) gene over-expressions; and (iv) gene dampenings.

This strategy ensures that reactions that are vital for either growth or target product production, or those that produce key metabolites, are not selected as potential knockouts. Conversely, reactions that (i) significantly divert carbon away from target production; and (ii) consume a metabolite known to promote flux loss from target production; are selected preferentially. Once the first knockout is predicted, the model is modified to block this reaction, and the same selection process is used to select the second reaction to delete. This method can be applied iteratively to predict a number of modifications that should enhance target production whilst maintaining growth.

All code was developed in Matlab to maintain compatibility with the COBRA Toolbox (Schellenberger et al., 2011), and is available in Supplementary Folder 1.

#### Metabolite Consumption Test (MCT)

**Figure 3:**
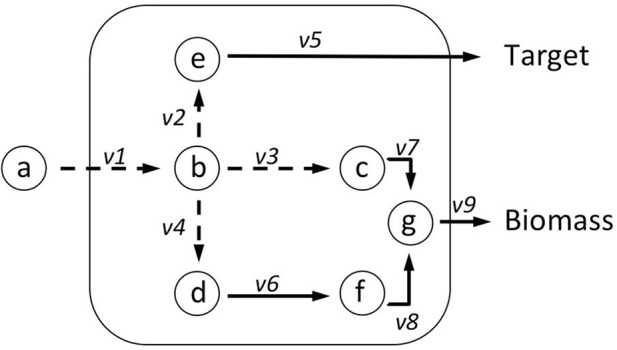
**Metabolite Consumption Test (MCT) identifies metabolites that are in the optimal target production pathway**. The test has two parts, first a *flux change* (*X_m_*) score is computed using Equation (1). Taking metabolite **b** as an example: *v1* produces **b** but is needed for both target and biomass production so weight (*w*_1_) = 0; *v2* consumes **b** but is needed for producing the target so *w*_2_ = 0; *v3* consumes **b**, so *w*_3_ = 1; *v4* consumes **b**, so *w*_4_ = 1. These values are multiplied by the absolute value of maximum flux calculated using FVA (*v^i^*_max_), and by a constant (*c*) = ±1 according to whether the reaction produces or consumes the metabolite. Where *X_m_* < 0 MCT = 1, where *X_m_* ≥ 0 MCT = 0. Reactions identified as suitable knockout targets using RobOKoD are sorted firstly by *R^i^_KOr_* and secondly by their MCT flag. This means that reactions with an equal *R^i^_KOr_* can be differentiated by a secondary sorting against whether they directly consume a metabolite that is important for the target production (see Table 2).

Metabolite Consumption Test (MCT) identifies metabolites within the optimal target production pathway that are also consumed to produce biomass. The MCT score is given in a two-step process. First *flux change* (X*_m_*) per metabolite (*m*) is calculated, then an MCT-value of 1 is given to all reactions that consume metabolites, denoted by a negative X*_m_*. X*_m_* is calculated according to Equation (1). For each metabolite that is featured in the optimal target producing pathway, for the example network in Figure 3, that would be metabolites **a**, **b**, **e**, all producing and consuming reactions are identified. Then per identified reaction, a unitary constant *c* is calculated which identifies the reaction as a producer (+1) or consumer (−1) of the metabolite during biomass production, thereby indicating whether there is a potential flux loss or gain from that reaction. Each reaction is then weighted (*w*) according to whether it is vital for both target and biomass (0); or potentially used (1), or not used (0) for biomass production. *v* is the maximum flux through the reaction during biomass production. All reactions that consume a metabolite *m* with a negative X*_m_*-value are flagged with a 1 in the corresponding column (see MCT column in Table 2).

(1) $$X_{m}=\sum_{i=1}^{n}c^{\left(i\right)}\cdot w^{\left(i\right)}\cdot v_{\text{max}}^{\left(i\right)}$$

**Table 2:** **Using the toy network presented in Figures 3, **4** we computed the MCT score and *R^i^_KOr_* of the intracellular reactions**.

**Flux**	**MCT score**	***R^i^_KOr_***
*v3*	1	0.8523
*v4*	1	0.8523
*v6*	0	0.8523
*v7*	0	0.8523
*v8*	0	0.8523
*v2*	0	0

*v3, v4, v6, v7, and v8 all have the same FVAp profiles and therefore R^i^_KOr_ scores. Of the top ranking reactions within this network v3 and v4 consume a metabolite that is important for target production. These reactions are then sorted as a higher priority within the equally ranked reactions to select as a knockout target*.

#### FVA reaction profile (FVAp)

Prior to FVAp, FBA is applied to predict the maximal theoretical yield of both biomass (*y*_bm_) and target product (*y_target_*). FVAp is then performed which computes the flux variability of each reaction: (1) at different percentage (0–100%) of *y*_bm_ whilst optimizing target product; and (2) at different percentage (0–100%) of *y_target_*, whilst optimizing biomass. By computing FVAp the flux capacity of each reaction is profiled over a range of target constraints. The key areas of interest are the extremes of target production, and biomass production. It can be seen in **Figure 5** that the first and last quartile of the *x* axis for all examples holds the key information from which beneficial genetic interventions can be inferred.

#### Knockout scoring

Knockouts were selected by computing a knockout ranking score. The ranking score is calculated for each reaction using FVAp at different percentage (0–100%) of *y*_bm_ whilst optimizing target product (red shaded area). Let us denote with (*v*_max_)*^target^*|_*p*_ and (*v*_min_)*^target^*|*_p_* the maximal and minimal flux, respectively of reaction *i* obtained through FVAp when requiring a percentage *p* of *y*_bm_ to be produced while maximizing for product. Likewise let the maximal and minimal flux of reaction *i* obtained through FVAp when requiring a percentage *p* of *y_target_* to be produced while maximizing for biomass be defined as (*v*_max_)*^biomass^*|*p* and (*v*_min_)*^biomass^*|*p*, respectively. It must be noted that the percentage *p* refers to either biomass or target product production requirement depending on the objective function.

A suitable knockout target displays the key characteristics shown in **Figure 5A**, where the first quartile of *x* axis 0-25% of *y*_bm_ (red shaded area) carries a lower *v^(i)^*_*max|target*_, than 75-100% of *y*_bm_, which shows that the reaction is required to carry a higher flux to sustain optimal biomass production. This characteristic is captured in Equation (2) (biomass reaction activation). A reduced variability in the fourth quartile also demonstrates a stronger constraint on the flux to produce *y*_bm_, this is captured in Equation (3) (product variability area). The final knockout scoring *R^i^_KOr_* for each reaction was computed according to Equation (4), which takes into account the features of both the biomass reaction activation and product variability area.

Biomass reaction activation:

(2) $$\sum_{p_{1}=75\%}^{100\%}{\left(v_{\text{max}}^{\left(i\right)}\right)}^{target}|p_{1}-\;\sum_{p_{2}=0\%}^{25\%}{\left(v_{\text{max}}^{\left(i\right)}\right)}^{target}|p_{2}$$

Product variability area:

(3) $$\sum_{p=75\%}^{100\%}{\left(v_{\text{max}}^{\left(i\right)}\right)}^{target}|p-{\left(v_{\text{min}}^{\left(i\right)}\right)}^{target}|p$$

(4) $$R_{KOr}^{i}=\;\frac{biomass\;reaction\;activation}{product\;variability\;area}$$

**Figure 4:**
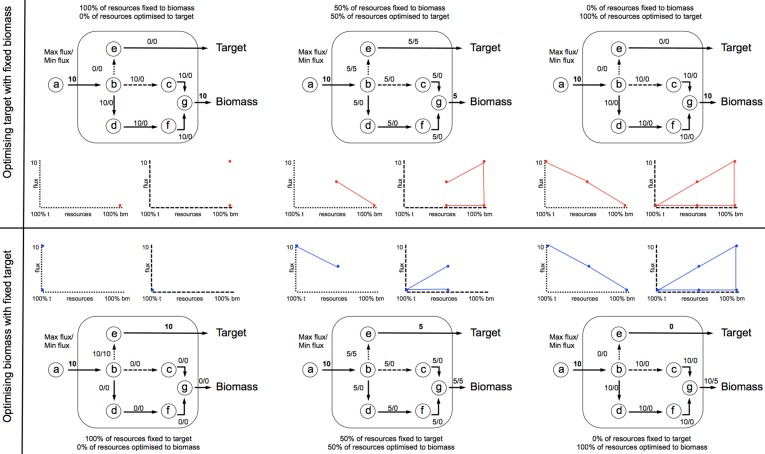
**FVA is performed to compute the flux variability of each reaction: (1) at different percentage (0–100%) of *y*_bm_ whilst optimizing target product; and (2) at different percentage (0–100%) of *y_target_*, whilst optimizing biomass**. Each iteration develops a profile of *v*^(*i*)^_min_ and *v*^(*i*)^_max_ across the range of flux space. In this example an input flux of 10 units through *v*_1_ is fixed, and the network optimized to situation 1 (top profile), or 2 (bottom profile).

Reactions that obtain a high *R^i^_KOr_*, are identified as a putative target for knocking out providing it is not a lethal target for the cell. Identified target reactions for knocking out are first ordered by *R^i^_KOr_*, before secondary sorting by MCT flags. An example of this sorting can be seen in Table 2 based on the toy network presented in Figures 3, 4.

#### Over-expression ranking

**Figure 5:**
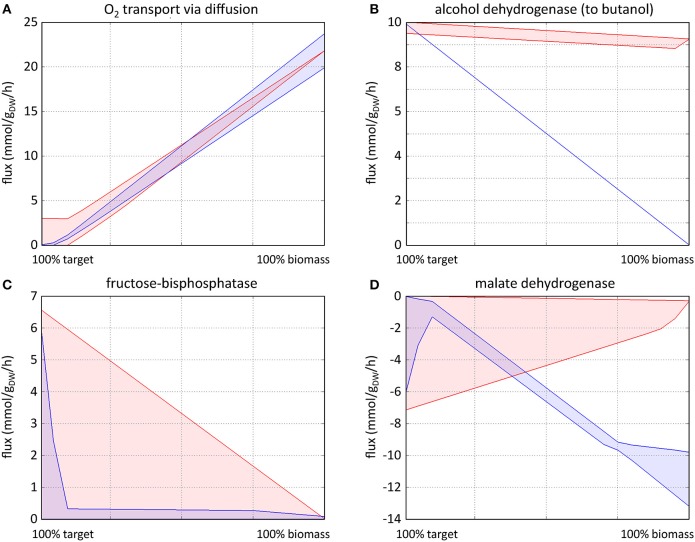
**Typical FVA profiles characteristic of knockout targets (A), strong overexpression targets (B), weak overexpression targets (C), and dampening targets (D)**. The red profiles show FVAp of each reaction at different percentages of (0–100%) of *y*_bm_ whilst optimizing target product. The blue profiles show FVAp at different percentages of (0–100%) of *y_target_* whilst optimizing biomass. Knockout targets **(A)** are identified using (75–100%) of *y*_bm_ (corresponding to the fourth quartile of *x* axis) with target optimization: where *v*^(*i*)^_max|*target*_ increases as *y*_bm_ increases, coupled with a reduced variability between *v*^(*i*)^_max|*target*_ and *v*^(*i*)^_min|*target*_. Strong overexpression targets **(B)** are identified using (0–25%) of *y*_bm_ optimizing target, and (75–100%) of *y_target_* optimizing biomass (corresponding to the first quartile of *x* axis), where *v*^(*i*)^_max|*target*_ (red) has a higher flux carrying capacity than *v*^(*i*)^_max_|*biomass* (blue), again with reduced variability between *v*^(*i*)^_max|*target*_ and *v*^(*i*)^_min|*target*_. Weak overexpression targets **(C)** show similar characteristics, with a smaller difference between *v*^(*i*)^_min|*target*_ and *v*^(*i*)^_min|*biomass*_ and a larger variability between *v*^(*i*)^_min|*target*_ and *v*^(*i*)^_min|*target*_. Profiles of dampening targets **(D)** are the reverse of overexpression targets.

The characteristics of a strong over-expression target can be seen in the lower quartile of *x* axis in Figure 5B, where at 0-25% of *y*_bm_ (red shaded area) *v*^(*i*)^_min|target_ has a higher flux capacity than 75-100% of *y_target_* (blue shaded area), *v*^(*i*)^_min|biomass_ (target extra flux, see Equation 5). A lower variability is also desirable for optimizing target subject to 0-25% of *y*_bm_ (target variability, Equation 6) as it ensures that the minimum flux the reaction can carry is close to optimum. The final ranking (*R^i^_OEx_*) is determined using Equation (7), where reactions with the highest *R^i^_Oex_* are the most likely over-expression targets. An example of a weaker over-expression target (corresponding to a lower *R^i^_OEx_*) is shown in Figure 5C, which illustrates an over-expression that will increase flux to both target *and* biomass. Negative *R^i^_OEx_* represent potential dampening targets (see Figure 5D), which display the opposite characteristics.

*Target extra flux*:

(5) $$\sum_{p_{1}=0\%}^{25\%}{\left(v_{\text{max}}^{\left(i\right)}\right)}^{target}|p_{1}-\;\sum_{p_{2}=75\%}^{100\%}{\left(v_{max}^{\left(i\right)}\right)}^{BM}|p_{2}$$

*Target variability*:

(6) $$\sum_{p=0\%}^{25\%}{\left(v_{\text{max}}^{\left(i\right)}\right)}^{target}|p-{\left(v_{\text{min}}^{\left(i\right)}\right)}^{target}|p$$

(7) $$R_{OEx}^{i}=\frac{target\;extra\;flux}{target\;variability}$$

### OptKnock and RobustKnock

The OptKnock algorithm (Burgard et al., 2003) is available in the COBRA Toolbox for Matlab, and RobustKnock algorithm is available as a Matlab script from the original paper (Tepper and Shlomi, 2010). Both are repackaged in Supplementary File 1 allowing for reproduction of the following results.

## Results

As a case study, RobOKoD was applied to design an *E. coli* strain with a reverse β-oxidation cycle for butanol production. These results can be recreated by unzipping the code in Supplementary File 1, and running the test script *iNS142_butanol.m* in Matlab [requires the COBRA Toolbox (Schellenberger et al., 2011), and if RobustKnock is to be tested, the Tomlab solver (Tomlab Optimization Inc., Västerås, Sweden)]. This test script runs RobOKoD over a maximum of five iterations of knockout scoring, implementing the highest scoring knockout, generating a results document and reaction FVA profile plots for each iteration in the directory *iNS142_butanol_results*, and outputting an updated SBML model in which the knockouts have been implemented. It subsequently runs over-expression ranking, again generating output in the *iNS142_butanol_results* directory. OptKnock and RobustKnock are then run in order to compare predictions from each method. Knockout scoring, over-expression rankings, and FVA profiles for all relevant reactions (such as those illustrated in Figure 3) can then be inspected manually.

MCT allows the identification of reactions which consume metabolites present in the optimal target production pathway that demonstrate flux loss toward biomass. These reactions are flagged in the listing of potential knockouts with a value of 1, allowing these reactions to be identified preferentially, out a set of reactions with the same knockout score. In this network, pyruvate was identified as a key metabolite where flux loss to biomass production could occur, 11 reactions were then identified that consume pyruvate.

FVA profiles representative of the different situations commonly encountered are shown in Figure 5. Knockout targets (Figure 5A) are identified based on fixed biomass optimal target FVAp (red profile). As the percentage of fixed biomass increases, the flux through the reaction increases to accommodate a higher biomass requirement, and the variability of the flux narrows. Strong overexpression targets (Figure 5B) show the opposite behavior of knockouts, whereby the flux through the reaction reduces as the percentage of fixed target is reduced as biomass is optimized (blue profile). Weak overexpression targets (Figure 5C) show similar characteristics, but are not required to carry a flux for the target to be optimized. Dampening targets (Figure 5D) are characterized by their ability to carry higher flux through a reactions at low percentage of fixed target with optimized biomass, than at both a high percent of fixed target and optimized biomass, and a low percent of fixed biomass and optimized target.

It is noted that some reactions obtain identical scores, hence their deletion are predicted to have the same impact on the system. This is for instance the case for two consecutive reactions of an unbranched, linear pathway. More generally, this is observed for the subsets of reactions that carry perfectly correlated fluxes (Heiner, 2009; Feist et al., 2010). A feature of RobOKoD is therefore its ability to identify such subsets of reactions. The corresponding knockouts are expected to result in a similar phenotype, hence the modification to perform for such subsets of reactions should be evaluated in the light of technical considerations. The most practical modifications should be selected, whilst the resulting strain should still be amongst the optimal producers.

For comparison purpose, the well-established algorithms OptKnock and RobustKnock were applied on the same model to predict the optimal strain for butanol production. For each method, the maximum number of modifications was fixed to five, since constructing such a strain can still be managed experimentally. The optimal producer strains predicted by each method are listed in Table 3 and are compared to the most efficient producer strain which has been experimentally validated (Dellomonaco et al., 2011). OptKnock and RobustKnock predicted strains that were theoretically unable to produce butanol during growth, and in the case of OptKnock, not viable for growth.

**Table 3:** **Gene modifications, based on the reactions predicted by the three computational methods, and their comparison with those successfully applied experimentally (Dellomonaco et al., 2011)**.

**Method**	**Gene modifications [Δ*gene*_(reaction)_]**
OptKnock	Δ*eutE*_(ACALD)_ Δ*nuoH*_(NADH16)_ Δ*amtB*_(NH4t)_ Δ*pflA*_(PFL)_ Δ*pitB*_(PIt2r)_
RobustKnock	Δ*lldP*_(D_LACt2)_ Δ*focA*_(FORti)_ Δ*pgi*_(PGI)_ Δ*satP*_(SUCCt2_2)_ Δ*sucD*_(SUCOAS)_
RobOKoD	Anoxic conditions_(O2t)_, Δ*pflA*_(PFL)_, Δ*eutE*_(ACALD),_ Δ*dld*_(LDH_D),_ *fadA*+, *yqeF*+
*Experimental*	RB02(fadR atoC(c) crp^*^ Δ*arcA* Δ*adhE* Δ*pta* Δ*frdA*) Δ*yqhD* Δ*eutE yqeF*+ *fucO*+

Table 4 compares the functionality modifications of the predicted *in silico* cells, and the experimental strain. It appears that RobOKoD automatically captures most of the functional modifications experimentally carried out. In particular, it predicted that fermentation pathways (*pfl, ldhA*) should be knocked out to avoid diversion of carbon and reduced cofactors toward by-products of poor interest. Moreover, by highlighting the competing interests of oxygen uptake pathway between the production of biomass and butanol, RobOKoD was able to indicate an anoxic condition change, similar to the experimental strain which knocked-out fumarate reductase and was grown under microaerobic conditions.

**Table 4:** **Functional similarities captured in the gene manipulations predicted by each method**.

**Gene**	**Function**	**OptKnock**	**RobustKnock**	**RobOKoD**
Δ*adhE*	Alcohol/acetaldehyde dehydrogenase			✓
Δ*pta*	Phosphotransacetylase			✓
Δ*frdA*	Fumarate reductase (respiration)		✓	
Δ*yqhD*	Alcohol dehydrogenase			✓
Δ*eutE*	Acetaldehyde dehydrogenase	✓		✓

In addition to the knockout predictions, RobOKoD was also able to predict over-expression and dampening targets. It predicted that enzymes catalyzing the reactions associated with the reverse β-oxidation cycle should be over-expressed, consistent with the experimental strain where the activity of transcriptional inhibitors of this pathway are dampened (fadR, atoC(c), crp^*^, and Δ*arcA strains*). Moreover, RobOKoD also predicts that a number of transport reactions (or rather *genes* encoding the relevant transport proteins) should be dampened, hence providing additional modifications that could enhance butanol production. These dampening predictions, less intuitive, were not carried out in the experimental strain and have not been experimentally verified.

Table 5 compares the molar production of butanol per mole of glucose uptake, when the objective of the cell is to optimize biomass. It shows that RobOKoD predicted the most successful butanol strain design, with molar ratio values similar to that achieved in the experimental strain. Neither OptKnock or RobustKnock predicted successful strains, and in the case of OptKnock, the strain was predicted to be no longer viable.

**Table 5:** **Molar ratio of glucose:butanol produced in predicted strains**.

**Method**	**Molar ratio (glucose:butanol)**
OptKnock	1:0
RobustKnock	1:0
RobOKoD	1:0.9
Experimental	1:0.8

The strain predicted by RobOKoD was developed iteratively by automatically knocking out the highest ranked suggested knockout target, that also was flagged by MCT as a potential route for flux loss from the butanol production pathway. This was to prevent selection bias for trialing its validity. It is strongly recommended to use the method more flexibly, looking at the FVAp graphs that are produced for the reactions, knowledge of the organism, and the scorings in order to decide on suitable knockouts.

## Discussion

These results illustrate two limitations of OptKnock and RobustKnock. First, the knockout predictions are deterministic, not ranked, and a unique set of knockouts is predicted. As shown by these results, different knockouts which may give similar phenotypes cannot be identified by these algorithms. With RobOKoD, a score is attributed to each modification, and one can readily check whether some modifications are expected to result in similar phenotypes and select those that can be more easily implemented experimentally. Secondly, OptKnock and RobustKnock are unable to predict over-expression or dampening strategies, which are of prime interest to increasing or decreasing flux down key pathways, respectively. However, it is argued that using a range of available techniques may help to build up a more comprehensive understanding of the system, and comparing the results obtained by different methods (e.g., Burgard et al., 2003; Choi et al., 2010; Tepper and Shlomi, 2010; Park et al., 2012) would be the most valuable strategy for designing producing strains.

It is also important to note that constraint-based modeling is not appropriate in all instances for prediction of suitable strains for target molecule production. FBA, a key method of assessing the functionality of a given strain, has the flaw whereby side reactions are not predicted to be carrying flux *in silico* as this would reduce the optimal resources that are routed to growth. An example being FBA run on yeast not producing ethanol under an intuitively appealing set of constraints (Westerhoff et al., 2009). This means that only solutions for target production pathways which are heavily coupled with growth can be identified. This is not an issue in most cases since a viable strain is desired but limits the applicability of this framework in particular cases, for example, when there is a need to decouple production from growth. It also means that the false negative rate for *in silico* strain predictions is high, with many successful laboratory strains not appearing so when translated to an *in silico* model. In future the field needs to look more toward different ways of predicting metabolic fluxes. Combining kinetic and stoichiometric models of the metabolic system (Chowdry et al., 2014) provides additional levels of constraints (including enzyme inhibition and activation) and is expected to improve the prediction of effective interventions. A longer term goal is therefore the production of detailed, large-scale kinetic models of the whole metabolic system (Stanford et al., 2013).

When running OptKnock and RobustKnock, it was clear that OptKnock was more user friendly, owing to it being made available in the COBRA Toolbox for Matlab and therefore applicable to a number of MILP (mixed integer linear programming) solvers. This was not the case for RobustKnock, which required a non-standardized model structure and the use of a specific solver, Tomlab, which has limited free access. An additional goal of designing RobOKoD was therefore to ensure its accessibility and robustness by reusing freely-accessible solvers, extensively validated COBRA Toolbox methods, and standardized model formats such as SBML.

A necessary future direction for both RobOKoD and existing tools such as OptKnock and RobustKnock will be to move to making predictions regarding knockouts, over-expressions, etc. at the level of the *gene*, rather than, as currently, at the level of the reaction. Due to the presence of both isoenzymes and promiscuous enzymes, it is clear that there is not a 1:1 mapping between gene and reaction. Consequently, manipulation of a given gene is likely to affect a number of reactions. Modification of this method to consider the gene-protein-reaction (GPR) relationships that are present in many genome-scale metabolic models will be a priority for future development.

To summarize, RobOKoD provides an additional tool to aid the task of designing strains for the (over)production of target products. It is able to predict and rank knockouts, over-expressions, and dampening targets. While predicting an optimized set of gene modifications to implement, unlike other methods, RobOKoD also provides lists of candidate modifications, along with graphical flux variability profiles, allowing the user to manually validate the set of predictions. Such a flexible approach—particularly when used in conjunction with other analysis methods mentioned previously—will allow for sensible gene manipulation approaches to be taken into the laboratory.

## Author contributions

NJS conceived the study, led the project, developed the method and the code, and wrote and led the writing of the manuscript. PM contributed to the method conception and development, and writing of the manuscript. NS contributed to the code development and the writing of the manuscript.

### Conflict of interest statement

The authors declare that the research was conducted in the absence of any commercial or financial relationships that could be construed as a potential conflict of interest.

## Reaction Abbreviations

**Table:**

**Model reaction ID**	**Reaction name**	**EC**
ACALD	Acetaldehyde dehydrogenase (acetylating)	1.2.1.10
er_027	Alcohol dehydrogenase (to butanol)	1.1.1.1
LDH_D	D-lactate dehydrogenase	1.1.1.27
NADH16	NADH dehydrogenase (ubiquinone)	1.6.5.3
NH4t	Ammonia reversible transport	n/a
O2t	O_2_ transport via diffusion	n/a
PFL	Pyruvate formate lyase	2.3.1.54
PIt2r	Phosphate reversible transport via proton symport	n/a
