# Supplementary material for: RobOKoD: microbial strain design for (over)production of target compounds
Source: Front Cell Dev Biol. 2015 Mar 24;3:17. doi: 10.3389/fcell.2015.00017 (PMC4371745; doi:10.3389/fcell.2015.00017)
Supplement: Supplementary file 1 [file supplementaryfolder1.ZIP › Supplementary File 1/ReadMe.docx]

RobOKoD:

This script reproduces results for RobOKoD, OptKnock, and RobustKnock. You will need cobra toolbox installed, and a MILP solver such as Gurobi. For RobustKnock TomLab solver is required.

To reproduce the results run iNS142_butanol.m
